# Water treadmill exercise reduces equine limb segmental accelerations and increases shock attenuation

**DOI:** 10.1186/s12917-019-2075-6

**Published:** 2019-09-13

**Authors:** Persephone Greco-Otto, Michael Baggaley, W. B. Edwards, Renaud Léguillette

**Affiliations:** 1 Department of Veterinary Clinical and Diagnostic Sciences, Faculty of Veterinary Medicine, University of Calgary, Calgary, AB T2N 4N1 Canada; 2 Human Performance Lab, Faculty of Kinesiology, University of Calgary, Calgary, AB T2N 4N1 Canada

**Keywords:** Equine, Accelerometry, Water treadmill, Sports medicine

## Abstract

**Background:**

Equine water treadmills (WTs) are growing in popularity because they are believed to allow for high resistance, low impact exercise. However, little is known about the effect of water height on limb loading. The aim of this study was to evaluate the effect of water height and speed on segmental acceleration and impact attenuation during WT exercise in horses. Three uniaxial accelerometers (sampling rate: 2500 Hz) were secured on the left forelimb (hoof, mid-cannon, mid-radius). Horses walked at two speeds (S1: 0.83 m/s, S2: 1.39 m/s) and three water heights (mid-cannon, carpus, stifle), with a dry WT control. Peak acceleration of each segment was averaged over five strides, attenuation was calculated, and stride frequency was estimated by the time between successive hoof contacts. Linear mixed effects models were used to examine the effects of water height, speed, and accelerometer location on peak acceleration, attenuation and stride frequency (*p* < 0.05).

**Results:**

Peak acceleration at all locations was lower with water of any height compared to the dry control (*p* < 0.0001). Acceleration was reduced with water at the height of the stifle compared to mid-cannon water height (*p* = 0.02). Water at the height of the stifle attenuated more impact than water at the height of the cannon (*p* = 0.0001).

**Conclusions:**

Water immersion during treadmill exercise reduced segmental accelerations and increased attenuation in horses. WT exercise may be beneficial in the rehabilitation of lower limb injuries in horses.

## Background

Equine gait is characterized by repeated impacts with the ground that are attenuated through active (muscle contraction) and passive (e.g. tendons, bones and cartilage) processes. Excessive rapid and repetitive loading of the limb has been associated with detrimental bone and joint changes, including degenerative joint disease, osteoarthritis, and fractures of long bones [1, 2]. Identifying interventions to limit overloading of the distal equine limb may reduce the risk of injury, and therefore extend the careers of these athletes.

Water treadmill (WTs) are increasingly being used for the rehabilitation and conditioning of equine athletes. Water treadmills are believed to provide a form of exercise that is high-resistance due to the viscosity of water, coupled with low impact due to buoyancy [3].

Previous studies have illustrated that the presence and height of water have a significant impact on the movement patterns of horses’ limbs. This is because water resistance and buoyancy increase as a function of water height [4, 5]. One kinematic study observed increased joint range of motion of horses walking on a WT at various water depths compared to no water [4]. The greatest amount of flexion observed in the carpal and hind fetlock joints occurred when water was at the height of the tarsal joint, while the greatest amount of flexion in the tarsal joint occurred when water was at the height of the tarsal and stifle joints [4]. Another study reported that stride frequency (SF) decreased and stride length (SL) increased with water heights at the carpus and ulna [6].

In humans, accelerometers have previously been used to characterize impact associated with locomotion. These studies include the transmission or attenuation of impact between anatomical locations, as well as the effect of changes in speed where increased speed leads to increased amplitude and frequency of limb shock, primarily due to increased stride length [7–11]. Accelerometers have also been used in equine research, mainly to investigate the effect of surface type [12, 13] or shoeing technique [14]. However, the effect of immersion in water on segmental acceleration and attenuation has not been examined in horses.

Therefore, the goal of this study was to assess the effects of speed and water height on peak segmental limb acceleration and shock attenuation during WT exercise. We expect that the presence of water will result in reduced peak acceleration at the limb, and that acceleration will progressively decrease with increased water height, while attenuation will increase. We also expect that acceleration will progressively increase with locomotion speed.

## Results

### Horses

Twenty-two Western performance Quarter horses [3 mares, 19 geldings; Median 6 years old (interquartile ranges: 5.5–10.5); Median 536.0 Kg (interquartile ranges: 489.5–558.5)] were enrolled in this study. Horses were all of similar sizes and proportions [mid cannon height – 29.2 cm (28.9–31.3 cm); carpus height – 42.5 cm (40.6–43.2 cm); stifle height – 88.9 cm (82.6–88.9 cm); withers height – 152.4 cm (152.4–155.9 cm); or, as a percentage of withers height: mid cannon – 19.2% (19.0–20.0%); carpus – 27.9% (26.7–27.7%); stifle – 58.3% (55.4–57.0%)].

### Peak accelerations

**Fig. 1:**
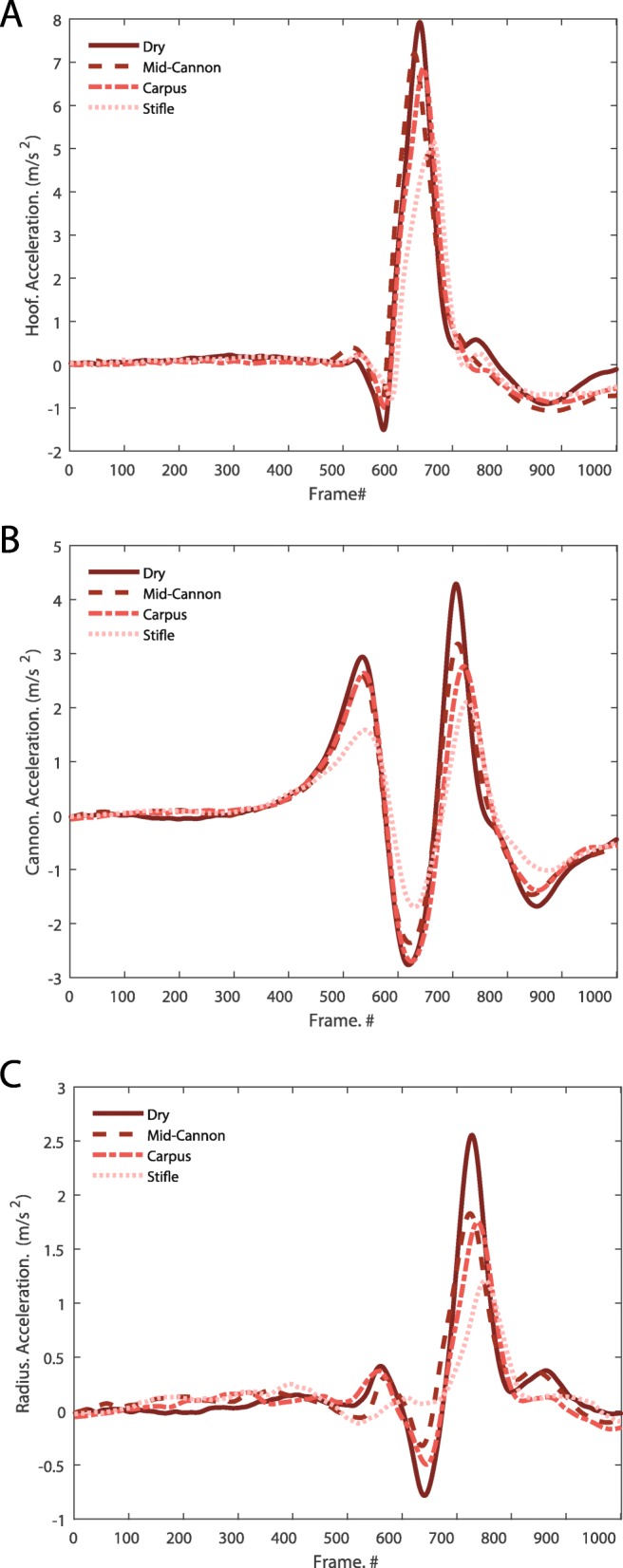
Group ensemble data of 21 horses for hoof (**a**), cannon (**b**), and radius (**c**) accelerometers during water treadmill exercise under four different water height conditions (dry, mid-cannon, carpus, stifle) at speed 2 (1.39 m/s). Individual strides were isolated for each accelerometer by extracting data 500 frames before, to 500 frames after, the peak cannon acceleration

**Fig. 2:**
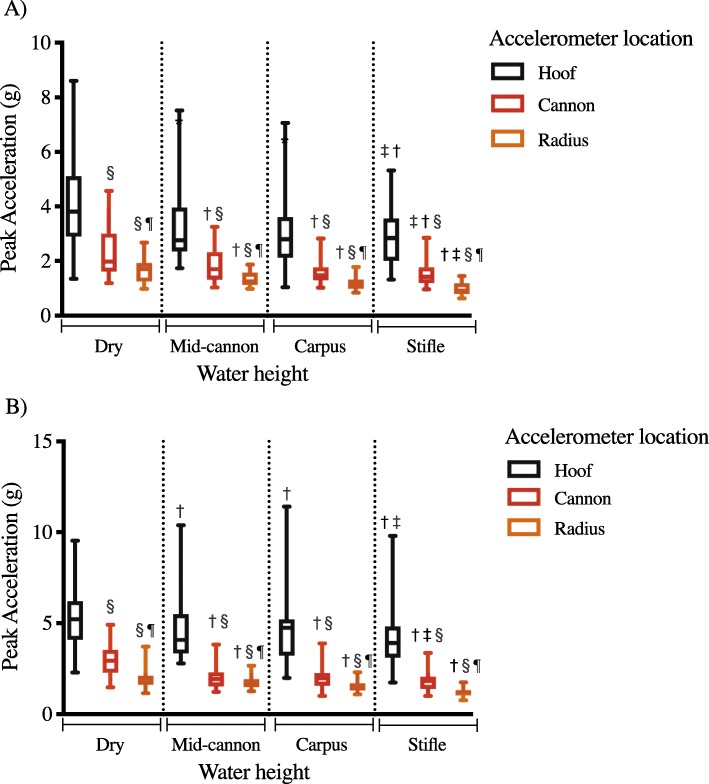
Impact peak accelerations of the hoof, cannon and radius at four water heights (dry, mid-cannon, carpus, stifle) during equine water treadmill exercise. Each parameter is represented by the median and interquartile range of 21 horses at each water height. **a** displays data collected at 0.83 m/s. **b** displays data collected at 1.39 m/s. † significantly different from dry values for the same accelerometer, ‡ significantly different from mid-cannon water height values for the same accelerometer, § significantly different from the hoof accelerometer for the same water height, significantly different from the cannon accelerometer for the same water height

Acceleration at the hoof, cannon and radius at all three water heights, as well as at the control (dry) condition, are shown in Fig. 1 and Table 1. At both speeds, peak acceleration was affected by both water height and accelerometer location (Fig. 2). Peak accelerations at the hoof, cannon, and radius were significantly lower with water of any height, compared to accelerations at the same location in the dry (control) condition (*p* < 0.0001 for all water heights). Peak acceleration was significantly reduced with water at the height of the stifle compared to mid-cannon water height (*p* = 0.04 for all locations). When comparing data within each water height, peak acceleration of the cannon and radius were lower than the peak acceleration of the hoof. Additionally, within each water height, peak acceleration of the radius was lower than that at the cannon.

**Table 1:** Accelerometry data collected from 21 horses during water treadmill exercise

	Dry (control)		Mid-cannon water height		Carpus water height		Stifle water height	
	Speed 1 (0.83 m/s)	Speed 2 (1.39 m/s)	Speed 1 (0.83 m/s)	Speed 2 (1.39 m/s)	Speed 1 (0.83 m/s)	Speed 2 (1.39 m/s)	Speed 1 (0.83 m/s)	Speed 2 (1.39 m/s)
Peak acceleration: Hoof (g)	3.81 (2.99–5.05)	5.24 (4.17–5.99)^d^ ^***^	2.77 (2.53–3.85)^a^ ^***^	4.11 (3.37–5.20)^a d^ ^***^ ^***^	2.80 (2.22–3.61)^a^ ^***^	4.76 (3.37–5.20)^a d^ ^***^ ^***^	2.85 (2.13–3.54)^a b^ ^***^ ^*^	3.93 (3.20–4.84)^a b d^ ^***^ ^*^ ^***^
Peak acceleration: Cannon (g)	1.99 (1.63–2.89)^c^ ^***^	2.95 (2.36–3.45)^c^ ^***^	1.72 (1.35–2.23)^a c^ ^***^ ^***^	1.95 (1.54–2.32)^a c^ ^***^ ^***^	1.49 (1.33–1.73)^a c^ ^***^ ^***^	1.99 (1.62–2.26)^a c^ ^***^ ^***^	1.43 (1.23–1.76)^a b c^ ^***^ ^***^ ^*^	1.85 (1.43–2.06)^a b c^ ^***^ ^***^ ^*^
Peak acceleration: Radius (g)	1.72 (1.28–1.91)^c e^ ^***^ ^**^	1.97 (1.72–2.14)^c e^ ^***^ ^**^	1.27 (1.17–1.56)^a c e^ ^***^ ^***^ ^**^	1.67 (1.51–1.93)^a c e^ ^***^ ^***^ ^**^	1.14 (0.99–1.29)^a c e^ ^***^ ^***^ ^**^	1.50 (1.33–1.70)^a c e^ ^***^ ^***^ ^**^	0.92 (0.80–1.16)^a b c e^ ^***^ ^*^ ^***^ ^**^	1.19 (1.05–1.31)^a b c e^ ^***^ ^*^ ^***^ ^**^
Frequency domain attenuation: Hoof-cannon (dB)	0.65 (−0.52–3.32)	0.00 (−1.70–4.36)	0.00 (− 1.17–2.91)	0.17 (−2.13–2.80)	0.23 (− 2.24–1.12)	−0.16 (− 2.07–1.14)	− 1.08 (− 1.76–0.14)^b^ ^***^	−0.92 (− 2.87–1.04)^b^ ^***^
Frequency domain attenuation: Cannon-radius (dB)	−7.51 [− 9.47-(− 6.74)]^f^ ^***^	−8.11 [− 9.01-(− 7.57)]^f^ ^***^	− 4.73 [− 6.97-(− 2.38)]^f^ ^***^	−6.03 [− 7.89-(− 4.51)]^f^ ^***^	−3.98 [− 7.01-(− 2.14)]^f^ ^***^	−7.75 [− 9.23-(− 5.97)]^f^ ^***^	−6.49 [− 9.01-(− 7.57)]^b f^ ^***^ ^***^	−8.25 [− 10.55-(− 5.70)]^b f^ ^***^ ^***^
Frequency domain attenuation: Hoof-radius (dB)	−8.19 [− 10.49-(− 6.37]^f^ ^***^	−8.74 [− 10.14-(− 5.04]^f^ ^***^	− 6.70 [− 9.80-(− 5.33)]^f^ ^***^	−8.37 [− 10.36-(− 4.44)]^f^ ^***^	−8.58 [− 10.12-(− 7.33)]^f^ ^***^	−9.34 [− 11.65-(− 6.14)]^f^ ^***^	−10.13 [− 12.51-(− 7.15)]^b f^ ^***^ ^***^	−9.47 [− 13.69-(− 8.88)]^b f^ ^***^ ^***^
Stride frequency (strides/min)	39.22 (37.53–42.68)	47.52 (44.16–48.85)^d ***^	37.27 (31.90–39.71)^a^ ^***^	40.48 (37.68–43.09)^a d^ ^***^ ^***^	34.25 (31.28–38.40)^a^ ^***^	39.92 (37.77–41.02)^a d^ ^***^ ^***^	35.55 (32.36–39.23)^a b^ ^***^ ^*^	38.15 (35.69–40.88)^a d^ ^***^ ^***^

All data is presented as median and interquartile range. ^a^significantly different from dry (control) values for the same accelerometer location and speed, ^b^ significantly different from mid-cannon water height values for the same accelerometer location, ^c^significantly different from the hoof accelerometer for the same water height and speed, ^d^ significantly different from speed 1 for the same water height and accelerometer location, ^e^significantly different from the cannon accelerometer for the same water height and speed ^f^significantly different from hoof-cannon attenuation for the same water height and speed. ***p < 0.001, **p < 0.01, *p < 0.05

Speed only had an effect at the hoof, with increased peak acceleration observed at the higher speed (*p* < 0.0001 for all water heights).

### Signal transmission

For a given speed, transmission from the hoof to the cannon, from the cannon to the radius, and from the hoof to the radius tended to increase with increasing water height (water at the height of the cannon vs. water at the height of the stifle) (*p* = 0.01) (Table 1). For a given water height and speed, transmission between the hoof and cannon was less than the transmission between the cannon and radius, and between the hoof and radius (*p* < 0.0001). Unlike water height, speed had no effect on attenuation under any conditions.

### Frequency-domain attenuation

Attenuation of acceleration between the hoof and cannon, cannon and radius, and hoof and radius tended to increase with increasing water height, such that water at the height of the stifle attenuated more impact than water at the height of the cannon (*p* = 0.0001) (Table 1). At all speeds and water heights, there is significantly less attenuation in the lower half of the limb (hoof-cannon) compared to the upper half (cannon-radius), as well as compared to the total attenuation of the limb (hoof-radius) (*p* < 0.0001 for all conditions). There is no difference in the attenuation occurring between the cannon and radius, compared to that occurring between the hoof and radius. There was a non-significant gain in signal power between the hoof and the cannon during exercise without water (dry control), and with water at the height of the mid-cannon and carpus for speed 1. Similarly, there was the same non-significant increase in signal power between the hoof and the cannon at speed 2, but only without water (dry) and with water at the height of the mid-cannon. Speed had no effect on attenuation under any conditions.

### Stride frequency

**Fig. 3:**
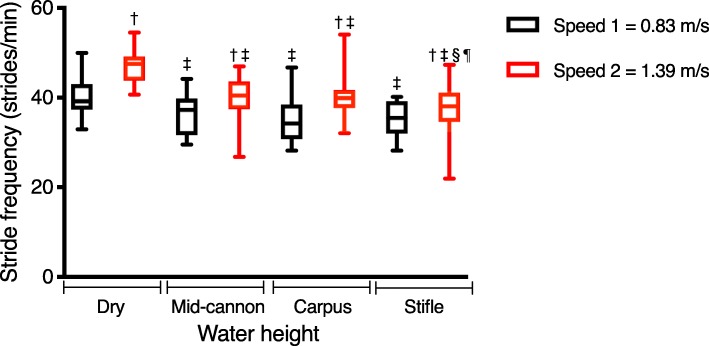
Stride frequency of 21 horses during water treadmill exercise at four water heights and two speeds. Each parameter is represented by the median and interquartile range. † significantly different from speed 1 for the same water height, ‡ significantly different from dry for the same speed, § significantly different from carpal water height for the same speed, ¶ significantly different from mid-cannon water height for the same speed

Both speed and water height affected stride frequency (Fig. 3, Table 1). For a given water height, increased speed resulted in a greater stride frequency. Stride frequency was lower at any height of water compared to dry (control) conditions (*p* < 0.0001) for any given speed. Stride frequency was lowest when working in water at the height of the stifle, compared to both mid-cannon and carpal water heights for speed 2 (*p* = 0.05, *p* = 0.03, respectively). Stride frequency was moderately positively correlated with peak acceleration (*r* = 0.62, *p* < 0.0001).

## Discussion

The present study used a randomized and controlled design to report for the first time the effects of WT exercise on horses’ limb acceleration and attenuation. WT exercise has been suggested as a means of conditioning and rehabilitating horses by harnessing the properties of water to reduce concussive forces, while achieving a mild to moderate workload.

Repetitive loading of the limb has been associated with the development of osteoarthritis in numerous species, including humans [15–19], laboratory animals [1, 20], and horses [21–23]. In vivo and in vitro experiments have shown that impact between the hoof and ground consist of high-frequency vibrations, a large percentage of which are attenuated between the wall of the hoof and the distal phalanx [24]. It has been argued that repetitive impulsive loading can lead to the development of microfractures of subchondral bone, eventually causing cartilage breakdown and joint degeneration [15]. Whereas many efforts have been made to limit these negative effects through various shoeing techniques [14] and changes in surface type [12, 25], little has been done to change horse training methods to reduce the risk of injury. In the present study, we have shown that WT exercise employing a high water height can effectively reduce acceleration and increase attenuation through the forelimb, potentially minimizing training-related injuries.

In a study that examined weight reduction of horses in a float tank, McClintock et al. found a 10.5% reduction with water at the level of the ulna, and a 31.3% reduction in bodyweight with water at the level of the point of the shoulder [5]. Unlike float tanks, during WT exercise horses are not fully buoyant and still make contact with the treadmill belt (i.e. are walking and not swimming). However, we observed a reduction of peak limb segmental acceleration with the addition of water – peak hoof acceleration was reduced by ~ 30% with water at the height of the stifle (the greatest water height tested) compared to the dry treadmill for the same speed. When dogs are placed in stifle height water on a WT, vertical ground reaction forces are reduced by 15% [26]. It is reasonable to assume similar results may be found with horses.

It has been well described that water treadmill exercise induces significant changes in gait pattern [4, 6]. Scott et al. observed a 9.2% decrease in SF from control (dry) to water at the height of the ulna [6]. Similarly, we found a 9.6% reduction in SF during exercise in stifle height water compared to the control (dry treadmill). It is thought that this change in stride mechanics, namely an increased flight arc, is due to a combination of vertical lift of the limb being facilitated by buoyancy while the motion of the leg in the sagittal plane is being slowed by water resistance [6]. The exaggerated arc increases flexion at the hip, stifle and hock [6]. Dogs exercising on WTs have also shown decreased SF and increased SL associated with increased stifle and hip flexion [27]. The correlation found between stride frequency and peak acceleration indicates that a reduced stride frequency is associated with reduced peak acceleration. Theoretically, there may be injury reduction benefits associated with horses taking fewer strides, where the acceleration of each stride is damped by water.

Rehabilitation can promote healing by increasing the flexibility of injured tissues, increasing bone and muscle strength, and restoring the range of motion of effected joints [28]. It has been indicated in humans [29] and small animals [30] that rehabilitative low-impact exercise allows for earlier use of the limb, resulting in increased range of motion, strength, and improved function, subsequently reducing the risk of re-injury [31]. WT exercise has recently been shown to be beneficial in the maintenance of horses with carpal osteoarthritis [32]. Indeed, King et al. reported that horses who were subjected to WT exercise had more symmetrical muscle recruitment and limb loading, improved synovial membrane integrity and greater joint range of motion, compared to horses that walked on a dry treadmill [32].

Since water height plays a significant role in the reduction of peak acceleration, it is necessary when using WTs for rehabilitation to choose an appropriate water height to target the injury location and stage of healing. When seeking to minimize limb acceleration, a high water height is indicated as peak accelerations decrease by 30% when water is at the height of the stifle. Furthermore, there is a 5% reduction of peak acceleration when water is increased from the height of the mid-cannon to the height of the stifle. However, in some cases, stifle water height cannot be tolerated due to the increased workload. We have previously shown that the greatest workload also occurs at the stifle water height [33] and that albeit a relatively low workload, at ~ 20% of V̇O_2_peak, it is sufficient to significantly improve fitness [34]. This suggests that the greatest workload and greatest dampening effects coincide with water at the height of the stifle. Therefore, every effort could be made to minimize impulsive forces through a combination of increased water height and reduced speed that is appropriate for the injury. This could help reach the goal of minimizing impact, while still allowing for a normal biomechanical function of the limb. Water at the height of the stifle will support the unsteady horse, and the confines of the treadmill reduce the risk of injury to horse and handler. As the primary injury begins to heal, a combination of different water heights may be used to increase loading of the limb to further the rehabilitation process. Water height can also be manipulated to increase joint range of motion by setting the height of the water to the level of the next joint above (proximal) the site of the injury [4]. As the injury heals and the horse is being prepared to re-enter normal work, a high water height should be used to maximize workload.

In the present study, increased water height resulted in decreased peak accelerations and increased attenuation. Lameness in sport horses is commonly associated with osteoarthritis type I of the metacarpalphalangeal joint [22, 23], and while the effect of different shoeing techniques has been shown to be negligible at this joint [24], a 30% reduction in peak acceleration at the hoof (as seen in this study with water) may have clinical significance. Therefore, this suggests that adding water may help to reduce the active and passive attenuation demands placed on the lamellae, muscles and joints of horses, aiding in the prevention or rehabilitation of musculoskeletal injuries.

## Conclusions

Water treadmills appear to be an effective tool to reduce acceleration of the equine limb during walking. Utilizing equine WTs with water at the height of the stifle reduced peak segmental acceleration and increased attenuation. Therefore, mild to moderate workloads [33] can be achieved in an environment that puts less stress on the lower limbs and may be beneficial in the rehabilitation of lower limb injuries in horses.

## Methods

### Horses

Privately owned Western performance horses (*n* = 22) were recruited from a rehabilitation and conditioning facility. Horses had no recent history of lameness, poor performance, or health issues, and all horses were screened for lameness by authors (PGO, RL) of this study. Horses were voluntarily enrolled in the study and owners completed a consent form. This study was approved by the University of Calgary Veterinary Sciences Animal Care Committee.

### Experimental protocol

All testing was conducted at a private rehabilitation and conditioning facility located in Alberta, Canada. Body weight was measured with a calibrated digital equine scale.^1^ Height of anatomical landmarks were measured with the horse standing square on a concrete surface from the ground with an inflexible measuring tape. These height markings were then transferred to the WT to ensure consistent filling during WT exercise. Horses were previously acclimated to the WT^2^ and were housed on-site for the duration of the study. Horses were considered acclimatized when they could calmly undergo all water height and speed combinations with a regular gait. Horses were not sedated at any time during acclimatization or data collection.

**Fig. 4:**
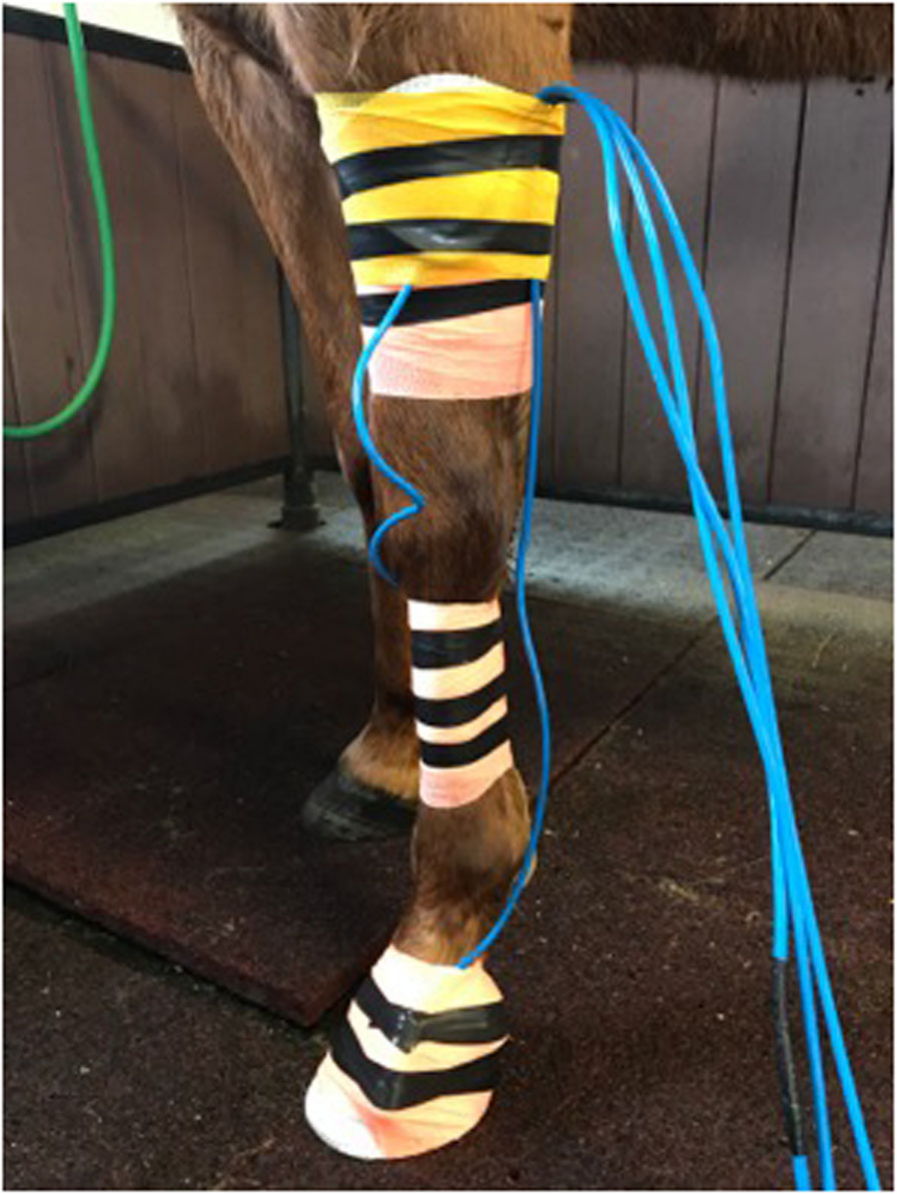
Application of three uniaxial, water submersible accelerometers to the left equine forelimb. Accelerometers are secured at the hoof (lateral), mid cannon (medial), and mid radius (medial) with adhesive wraps

Uniaxial accelerometers^3^ (±80G) were placed at the hoof (lateral), mid-cannon (medial), and mid-radius (medial) of the left forelimb (Fig. 4). Positions were chosen such that the accelerometers were secured over areas with limited soft-tissue between skin and bone (or on the hoof) to minimize motion artifact. Accelerometers were mounted so that the axis of measurement was in line with the long axis of each segment. Accelerometers were affixed to each segment using medical adhesive bandage,^4^ bandaging tape^5^ and electrical tape for a waterproof system. Horses were first trotted in hand for two passes of 250 m (500 m total) to allow for movement to normalize from the tactile stimulation of the accelerometers and adhesive bandages [35–38].

For testing conditions, horses walked at two speeds (0.83 and 1.39 m/s) and three water heights (mid cannon, carpus, and stifle) with a dry WT acting as a control, in a randomized order. Walking speeds were chosen so that they were comfortable for all horses. Once on the WT, horses were given 1 min to acclimatize to each speed/water height condition, followed by 1 min of data collection. Synchronized accelerometry data were collected at 2500 Hz using a WinDAQ ADC.^6^

### Data processing

Data were processed using a custom Matlab^7^ script. Individual strides were identified using peaks of the cannon accelerometer. To do this, the cannon accelerometer signal was filtered using a low-pass 4th order Butterworth filter with a cut-off frequency of 10 Hz. Peaks in the cannon accelerometer were identified as the beginning of a new stride if they exceed 0.5G and occurred more than 50% of the average stride time after the previous peak. Average stride time was determined using auto-correlation of the filtered signal [39]. Individual strides were isolated for each accelerometer by extracting data 500 frames before, to 500 frames after, the peak cannon acceleration. Peak acceleration for each segment was subsequently determined. Raw accelerometry data was filtered using a 4th order low-pass Butterworth filter with a cut-off frequency that retained 98% of the signal power as determined from spectral analysis (range = 70–300 Hz). A 0.2 ms search window was created around the identified limb contacts for each segment, and the peak acceleration was identified for the hoof, cannon, and radius. Signal transmission was calculated as:

$$ {\mathrm{Accel}}_{\mathrm{Transmission}}=\left(\frac{{\mathrm{Accel}}_{\mathrm{proximal}}}{{\mathrm{Accel}}_{\mathrm{distal}}}\right)\ast 100 $$

**Fig. 5:**
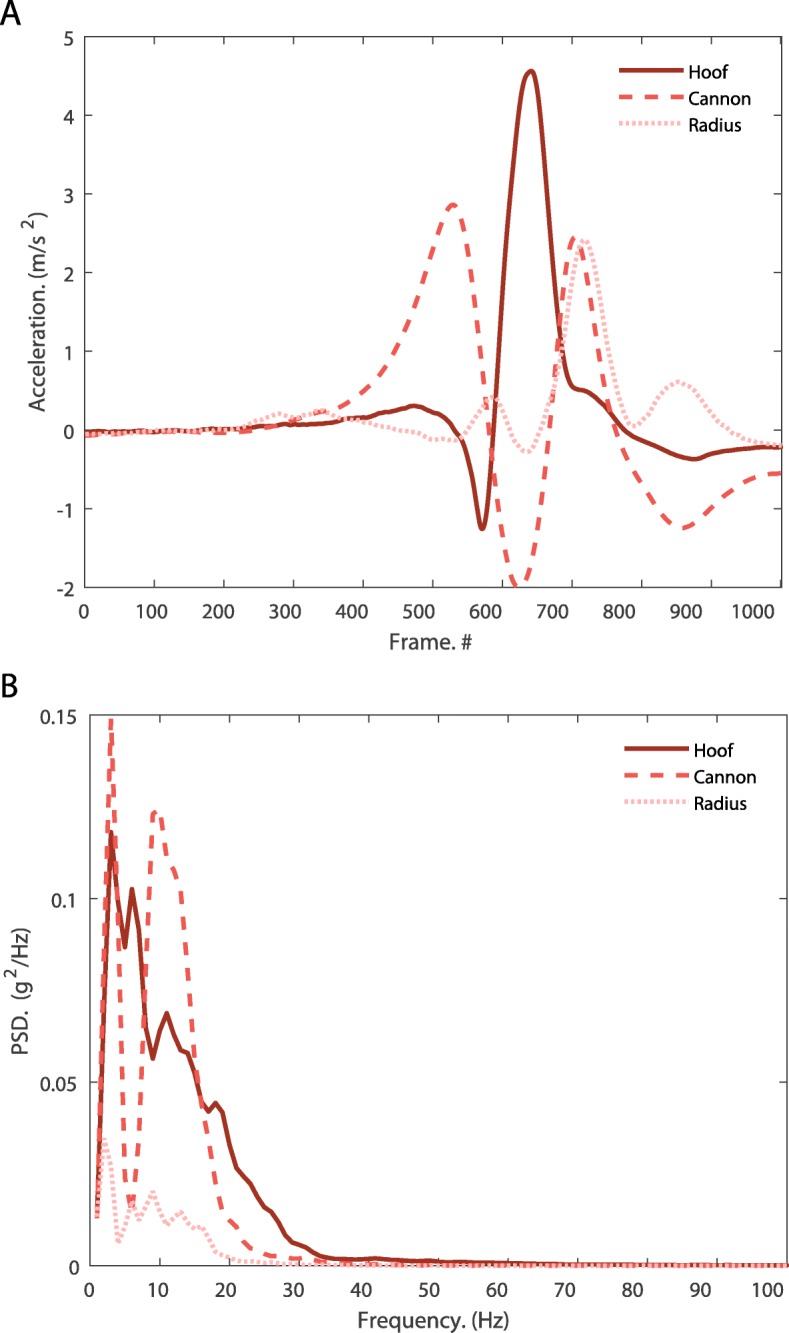
**a** Ensemble time-series data of segmental accelerations for all horses walking at speed 2 (1.39 m/s) with no water. Individual strides were isolated for each accelerometer by extracting data 500 frames before, to 500 frames after, the peak cannon acceleration. **b** Ensemble power spectral density of each accelerometer for all horses during water treadmill exercise at speed 2 without water

Where Accel_transmission_ is the ratio of the peak acceleration of the proximal segment and the distal segment expressed as a percentage for each stride. Frequency domain attenuation was calculated within the same 0.2 ms search window, from the power spectral density (PSD) curve with a frequency bin resolution of 1 Hz. Impact attenuation between the hoof – cannon, cannon – radius, and hoof – radius at each frequency within the 11–20 Hz frequency range was quantified with a transfer function:

$$ {\mathrm{TF}}_{\mathrm{i}}=10\kern0.5em {\log}_{10}\left(\frac{{\mathrm{PSD}}_{proximal\kern0.5em segment,\kern0.5em i}}{{\mathrm{PSD}}_{\mathrm{distal}\kern0.5em \mathrm{segment},\kern0.5em \mathrm{i}}}\right) $$

where TF_i_ is the attenuation (in dB) between the distal segment and proximal segment power spectral densities for the i-th frequency bin within the 11-20 Hz frequency range. An average TF within the 11-20 Hz range was subsequently determined. The 11-20 Hz frequency range was chosen as it represents the second major frequency domain component of the cannon accelerometry signal (Fig. 5), which has been suggested to represent frequency components due to impact [7].

Stride frequency was calculated as one over the time between each successive hoof impact for each of the five strides analyzed. This was then extrapolated to estimate the average stride frequency over 60 s. Peak acceleration of each segment, time-domain attenuation, frequency domain attenuation, and stride frequency were identified for five strides and averaged to give a single value for each condition.

### Statistical analysis

Linear mixed effects models were used to examine the effects of water height, speed, and accelerometer location (as fixed effects) on peak acceleration, attenuation and stride frequency (as outcomes), after accounting for the nested data structure from horses (as a random effect). The assumptions of normality and equal variance were assessed for these models. Analyses were performed using R version 3.3.2, and ‘nlme’ package version 3.1 was used for linear mixed effects models analysis. Associations between stride length and peak accelerations were examined using a Spearman correlation. Statistical significance was set at *p* ≤ 0.05 for all tests. All values are reported as median and interquartile range (IQR) to accommodate non-normal data.

## Data Availability

The datasets generated and/or analysed during the current study are not publicly available due to confidentiality agreements with the owners of the horses but are available from the corresponding author on reasonable request.

## Abbreviations

DT: Dry treadmill
SF: Stride frequency
SL: Stride length
V̇O_2_max: Maximum oxygen consumption
WT: Water treadmill

## Footnotes

1 Tokyo, Horse Weigh, Powys, Wales.

2 Horse-Trainer Water, Horse Trainer, Bygmarken, Denmark.

3 HS-4200205401, Hansford Sensors Ltd., Wycombe, England.

4 Lightplast Pro, BSN Médical, Laval, Québec.

5 Vetrap, 3 M, Maplewood, Minnesota.

6 WinDaq ADC, DATAQ Instruments, Akron, Ohio.

7 MatLab, Mathworks, Natrick, Massachusetts.
